# The role of copy number variants in the genetic architecture of common familial epilepsies

**DOI:** 10.1111/epi.17860

**Published:** 2024-01-20

**Authors:** Epi4K Consortium

**Affiliations:** * Consortium members listed in the appendix

## Abstract

**Objective:**

Copy number variants (CNVs) contribute to genetic risk and genetic etiology of both rare and common epilepsies. While many studies have explored the role of CNVs in sporadic or severe cases, fewer have been done in familial generalized and focal epilepsies.

**Methods:**

We analyzed exome sequence data from 267 multiplex families and 859 first-degree relative pairs with a diagnosis of genetic generalized epilepsies (GGE) or non-acquired focal epilepsies (NAFE) to predict CNVs. Validation and segregation studies were performed using an orthogonal method when possible.

**Results:**

We identified CNVs likely to contribute to epilepsy risk or etiology in the probands of 43/1116 (3.9%) families, including known recurrent CNVs (16p13.11 deletion, 15q13.3 deletion, 15q11.2 deletion, 16p11.2 duplication, 1q21.1 duplication, and 5-Mb duplication of 15q11q13). We also identified CNVs affecting monogenic epilepsy genes, including four families with CNVs disrupting the *DEPDC5* gene, and a *de novo* deletion of *HNRNPU* in one affected individual from a multiplex family. Several large CNVs (>500 kb) of uncertain clinical significance were identified, including a deletion in 18q, a large duplication encompassing the *SCN1A* gene, and a 15q13.3 duplication (BP4-BP5).

**Significance:**

The overall CNV landscape in common familial epilepsies is similar to that of sporadic epilepsies with large recurrent deletions at 15q11, 15q13, and 16p13 contributing to 2.5–3% of families. CNVs that interrupt known epilepsy genes, and rare, large CNVs were also identified. Multiple etiologies were found in a subset of families, emphasizing the importance of genetic testing for multiple affected family members. Rare CNVs found in a single proband remain difficult to interpret and require larger cohorts to confirm their potential role in disease. Overall, our work indicates that CNVs contribute to the complex genetic architecture of familial generalized and focal epilepsies, supporting the role for clinical testing in affected individuals.

## INTRODUCTION

Both rare and common genetic variants contribute to the etiology of epilepsies including genetic generalized epilepsies (GGE), non-acquired focal epilepsies (NAFE), and the more severe and rare developmental and epileptic encephalopathies (DEE). Significant advances have been made in uncovering *de novo* pathogenic variants in single genes as an important genetic cause of DEE ^1,2^. In the more common epilepsies, GGE and NAFE, single-gene causes are rare, and the genetic architecture is thought to be more complex ^3^. Exome sequence analysis in a large cohort of NAFE and GGE found an enrichment of ultra-rare single nucleotide variants in unrelated, affected individuals with a family history of GGE or NAFE ^4^, and genome wide-association studies have identified common variants associated with GGE, focal epilepsy or both ^5^.

Copy number variants (CNVs) have long been known to contribute to the genetic etiology of epilepsy. In the DEEs, *de novo* pathogenic CNVs account for 5–10% of cases ^6,7^. Studies of individuals with epilepsy plus other features including intellectual disability or autism found likely pathogenic CNVs in 10–13% of cases ^8–10^. In the more common epilepsies, recurrent CNVs including deletions at 15q11, 15q13 and 16p13 can increase the risk of developing epilepsy ^11–13^, but few studies have examined the contribution of rare CNVs to GGE and NAFE in rare families with multiple affected individuals. Here, we use exome sequence data to investigate the contribution of CNVs to the genetic architecture of familial GGE and NAFE using two cohorts: (i) 289 families with three or more affected individuals (multiplex families) and (ii) 859 individuals with GGE or NAFE and an affected first-degree relative (pairs).

## METHODS

### Cohorts

#### Multiplex Families:

566 individuals from 289 unrelated families were included (Supplementary Table 1); families were from cohorts at the University of Melbourne, Columbia University, University of Montreal, Swansea University, The Royal College of Surgeons, Ireland and the Epilepsy Phenome/Genome Project ^14^ assembled as part of Epi4K as previously described ^15^. Inclusion criteria included: at least three affected individuals with non-acquired epilepsy, two or more of whom had a diagnosis of GGE or NAFE. The families and affected individuals were previously described in detail ^15^. Depending on family structure and available samples, exome sequencing was performed for a minimum of 1 and a maximum of 5 individuals per family.

#### Pairs:

859 first-degree affected relative pairs (Supplementary Table 1), each with non-acquired generalized, focal or mixed epilepsy, were recruited through the Epilepsy Phenome/Genome Project between November, 2007 and August, 2013 ^14^. Each pair included one affected individual, designated as the proband, and their affected full sibling, parent, or child for a total of 1718 affected individuals. Exome sequencing was performed on one individual from each pair (designated the proband); SNP genotyping (HumanCore12, Illumina) was performed on both individuals from each pair.

This study used de-identified data from prior studies and did not require additional ethics approval. Each of the contributing studies was approved by the institutional review boards at the participating sites.

#### CNV prediction and validation

Exome data were generated as previously described ^4^ and analyzed with CoNIFER, a CNV prediction algorithm that relies on exome sequencing ‘read depth’ to predict deletion or duplication^16 17^ (Supplementary Tables 2, 3). Putative CNVs were filtered to include those that contained >4 exons and <70% overlap with segmental duplication and were predicted to be present in <5% of the cohort. Because CoNIFER is a prediction algorithm, we used chromosome arrays (custom oligonucleotide array comparative genomic hybridization (Agilent, Santa Clara CA) or SNP array data (HumanCore-12) to validate high-priority CNVs when DNA was available. Segregation testing was performed when DNA was available from additional family members (first-degree relative in the pairs or available family members in multiplex families). CNVs that were present in both affected individuals in the pairs cohort are referred to as concordant. **Pairs:** Exome analysis predicted 683 rare CNVs in 454 probands from the pairs. We performed validation studies (SNP arrays or aCGH) based on DNA availability and array coverage (Supplementary Table 2; completed for 629 predicted CNVs in 427 individuals). **Families:** In the multiplex families, 63 individuals from 45 families were removed from further analysis due to an excess of CNV calls (>50 per individual, which usually indicates poor DNA quality), leaving 503 individuals from 267 families. After filtering for rare CNVs, 1132 CNVs in 374 individuals were predicted (range 0–42, mean 8.8 per individual; Supplementary Table 3). Breakpoints for *DEPDC5* CNVs were determined by targeted long-read sequencing (T-LRS) on the Oxford Nanopore Technologies (ONT) platform or breakpoint PCR followed by Sanger sequencing.

#### Targeted long-read sequencing

Two CNVs in DEPDC5 were validated using T-LRS on the ONT platform. Briefly, DNA for sequencing was sheared to an average length of 10 kb using a Covaris g-TUBE by centrifuging at 4,000 RPM for 1 minute. Libraries for sequencing were prepared using the ONT SQK-LSK110 ligation kit following the manufacturer’s instructions except that the ligation reaction was allowed to proceed for 30 minutes instead of 10. DNA libraries were quantified using a qubit fluorometer and approximately 400 ng of DNA was loaded onto a ONT GridION R9.4.1 flowcell and run for at least 48 hours using Adaptive Sampling with a target region of chr22:30,000,000–33,000,000 (GRCh38). Sequencing data was basecalled using Guppy 5.0.7 using the superior model (ONT), FASTQ files were aligned to GRCh38 using minimap2 ^18^. CNVs were visually evaluated using IGV ^19^.

## RESULTS

In total, among our final included cohorts of 859 pairs and 267 multiplex families (total 1116 pedigrees), we identified CNVs likely contributing to epilepsy risk in the probands of 43 (3.9%) families and duos(Table 1; Table 2). These included recurrent CNVs at six loci that are known to be risk alleles for epilepsy and neurodevelopmental disorders (n = 30); CNVs that affected known epilepsy genes (n = 5); and CNVs larger than 500 kb (n = 8). These three categories of CNVs are detailed below. Segregation testing of additional family members highlighted the complex role of these CNVs to epilepsy risk: in total, 24/34 affected relatives who were available to be tested carried the CNV identified in the proband; in 5 families, the proband’s CNV was inherited from an unaffected parent.

### Pairs:

We predicted one or more rare CNVs in 454 probands; additional DNA for CNV validation by chromosome array was available for 427 probands. In those 427 probands, we confirmed 484/629 (77%) predicted CNVs. We next asked whether the validated CNVs were present in the affected first-degree relative. DNA was available to perform validation studies for 346/484 CNVs; 141 (41%) were present in both affected family members while 205 (59%) were present in the proband only. Of the 141 pairs who shared one or more CNVs, 60 (43%) had the same phenotypes (34 GGE, 15 NAFE, 11 mixed/unclassified epilepsy). As shown in Table 1, a subset of CNVs (n=29) are likely associated with epilepsy risk; the remainder will require additional data to determine disease association.

### Families:

In the families, we prioritized rare CNVs that were recurrent and known to be associated with neurodevelopmental disorders or were recurrent in our cohort, and rare CNVs that involved one or more genes associated with epilepsy or related neurodevelopmental disorders (Supplementary Table 4). Using these criteria, we identified 25 high-priority CNVs in 22 families (Supplementary Table 3) where DNA was available from additional family members for validation; we confirmed 12 (48%) CNVs in 11 families by chromosome array (Table 2). DNA was not available for three individuals predicted to have a large, recurrent deletion at 15q11 (n=2) or 15q13 (n=1). All predicted CNVs at these loci were validated in other individuals, so we considered these highly likely to be real; furthermore, in two cases the CNV was also predicted and validated in a family member. In total, 14 families had one or more validated, likely disease-associated CNV (Table 1; segregation results in Supplemental Figures).

Aside from known recurrent CNVs, none of the validated, concordant CNVs was found in multiple unrelated pairs or families.

### Recurrent CNVs associated with epilepsy risk

We identified recurrent CNVs associated with risk for epilepsy or related neurodevelopmental disorders in 23/859 (2.7%) probands from the pairs cohort and in 7 unrelated individuals from 7 of the 267 (2.6%) multiplex families (Table 1). Recurrent CNVs included 16p13.11 deletion (n=9), 15q13.3 deletion (n=5), 15q11.2 deletion (n=11), 16p11.2 duplication (n=2), 1q21.1 duplication (n=2), and a 5-Mb duplication of 15q11q13 (BP2–3). In 15/23 pairs, both affected individuals had the recurrent CNV, while in the remaining only one was a carrier. Similarly, in none of the families did all affected individuals have the recurrent CNV (Supplemental Material).

### Likely pathogenic CNVs involving known epilepsy genes

We identified 4 probands (3 pairs, 1 family) with CNVs involving the *DEPDC5* gene including 3 intragenic deletions and 1 intragenic duplication predicted to disrupt the DEPDC5 protein. In each of the three pairs, the affected first-degree relative also had the *DEPDC5* CNV; in the family, the proband and affected sibling inherited the CNV from their unaffected father. Sequence analysis of the CNV breakpoints involving *DEPDC5* revealed *Alu* elements at or near (21 bp) the breakpoints in 3 of 4 cases (Supplementary Table 5). A deletion encompassing *HNRNPU* was identified in the proband of a multiplex family; segregation studies showed that the deletion occurred *de novo*, suggesting that other genetic factors must be contributing to the epilepsy in the extended family. We also identified CNVs involving *KCNT1*, *PLCB1*, and *TCF4*, though the clinical significance of each was not clear.

### Large CNVs of uncertain significance

Because large CNVs are more likely to be pathogenic, we attempted to validate predicted CNVs larger than 500kb in families with available DNA. We identified a 7.5-Mb deletion of 18q in a proband with GGE; further analyses revealed that the unaffected father was mosaic for the deletion, and the affected mother did not have the deletion. The proband in another family harbors a 558kb duplication that encompasses the *SCN1A* gene; the duplication was inherited from the unaffected father and was not present in an affected maternal half-sibling. A recurrent duplication of 15q13.3 (BP4-BP5; reciprocal to 15q13.3 deletion) was identified in two affected siblings in one family; in another family, the same duplication was present in an unaffected parent but absent from the affected child.

## DISCUSSION

We investigated the role of rare CNVs in familial GGE and NAFE. Collectively, rare CNVs are common; on average, we predicted ~2 rare CNVs per exome. However, given that most CNVs that we identified (and validated) are present in a single family, with many found in only a single affected individual, determining which CNVs cause or increase risk for epilepsy is challenging. We therefore focused our studies on CNVs that were recurrent, large, or encompassed known epilepsy genes. Larger cohorts will be required to confirm the role of other rare CNVs in conferring epilepsy risk.

Our study confirms the role of large recurrent CNVs as risk factors and identifies several CNVs that include part of all or part of a known epilepsy gene. A number of recurrent CNVs have been associated with variable neurodevelopmental phenotypes, including epilepsy ^20^. Most notably, deletions of 15q13.3 confer a high risk of epilepsy ^12,13^. As in previous studies, we find that 2–3% of the families in both arms of our study have a recurrent CNV previously found to be associated with risk of a neurodevelopmental disorder. This frequency is similar to that seen in cohorts of sporadic epilepsy cases ^13,21^, which suggests that these CNVs do not disproportionately contribute to familial occurrence of epilepsies. Consistent with their role as risk factors ^22^, of the 23 probands from the pairs group with one of these CNVs, only 15/23 (65%) of affected first-degree relatives had the same CNV; in the families, the recurrent CNVs did not fully segregate with phenotype in any family. Analysis of other variants in the exomes of affected individuals may reveal additional genetic factors contributing to phenotypes in these familial cases. We identified two individuals with duplications of 15q13.3, reciprocal to the risk-associated deletion. The clinical significance of this duplication remains unclear, both in the literature^23^ and in our study.

We identified several CNVs involving known epilepsy genes, with four families harboring intragenic deletions or duplications disrupting the *DEPDC5* gene. Loss-of-function variants in *DEPDC5* cause familial epilepsy with variable foci (FEVF) which, in small families, typically presents with a mixture of frontal lobe and/or temporal lobe epilepsies though other focal epilepsies and epileptic spasms are also recognized ^24–26^. Furthermore, in an exome sequencing study comparing 525 individuals with familial NAFE to 3877 unaffected controls, ultra-rare variants in *DEPDC5* were significantly enriched in the affected cohort ^4^. Though few intragenic CNVs in DEPDC5 have been reported ^27,28^, our data suggest that deletion/duplication studies should be routinely incorporated when investigating *DEPDC5*. The *DEPDC5* gene is highly enriched for *Alu* repeats (34%) compared to the genome average (10%) ^29^; our sequence analysis of the breakpoints revealed *Alu*-mediated recombination likely contributes to the generation of intragenic *DEPDC5* CNVs, as has been shown for intragenic deletions of *ALDH7A1* ^30^. In one large family with multiple affected individuals (family cif), we identified *de novo* deletion encompassing the *HNRNPU* gene in a single individual. Upon review, her phenotype is more severe and is consistent with *HNRNPU*-associated epilepsy, which is likely genetically distinct from the epilepsy in other family members. This family highlights the fact that multiple genetic (or other) etiologies may be present in a single family.

**Figure 1:**
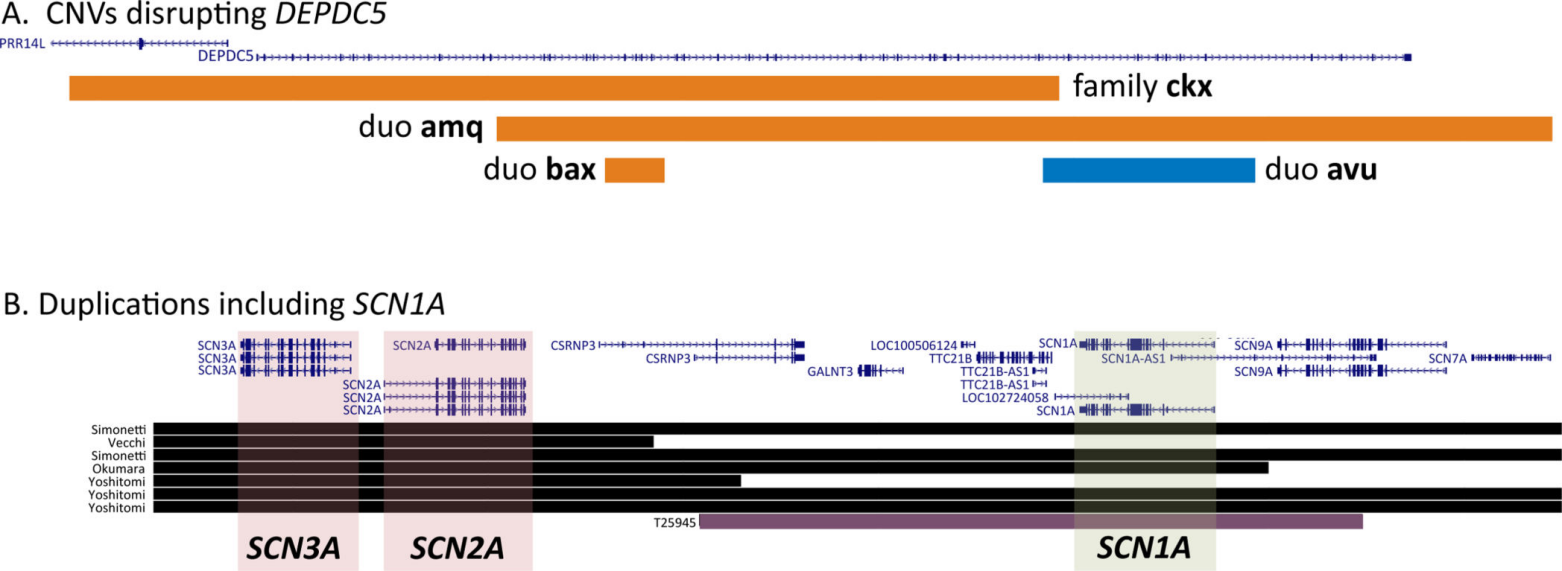
CNVs involving *DEPDC5* (A) and *SCN1A* (B) presented in the UCSC Genome Browser (hg19). For *DEPDC5*, 3 families harbored a partial deletion (orange) and one a partial duplication (blue) of the gene. For *SCN1A*, the green bar represents the duplication encompassing all of *SCN1A* (green shading) and surrounding DNA, though it does not extend to *SCN2A* or *SCN3A* (red shading). Black bars indicate CNVs from the literature involving *SCN2A* and *SCN3A*. Extent of the colored bars indicate extent of the CNV.

One proband has a duplication encompassing the entire *SCN1A* gene, a well-established cause of Dravet syndrome and related epilepsies including GEFS+. Although duplications of 2q24 have been associated with DEE, unlike the duplication in this proband all previously reported DEE-associated duplications reported to date that include *SCN1A* also include the *SCN2A* and *SCN3A* genes (FIGURE 1) ^31–34^. The duplication in the proband in our study, was inherited from an unaffected father, and the proband’s phenotype is much less severe than that of previously described individuals with 2q24 duplications. Overexpression of wild-type SCN1A protein in mice induced by an antisense oligonucleotide ^35^ does not cause a seizure phenotype or increased mortality; these data combined with the lack of phenotype in the carrier father suggest that the duplication in our proband could be benign. Although large CNVs are more likely to be pathogenic, we also identified a 7.5 Mb deletion of 18q22 in one individual that was inherited from an unaffected father, who is mosaic. Although deletions involving terminal 18q are associated with syndromic features ^36^, interstitial deletions of 18q22 have rarely been reported, leaving the significance in this case unclear.

Our study confirms that CNVs contribute to the complex genetic architecture of epilepsies in families, with recurrent CNVs playing a prominent role as risk factors with incomplete penetrance. We show that intragenic CNVs involving DEPDC5 may be more common than previously realized. CNV testing, whether through exome analysis or orthogonal platforms, should be incorporated into the genetic evaluation of familial epilepsy. Of course, there are limitations to this study. We used exome data for CNV prediction, which will not detect CNVs in intronic or intragenic regions, so we may have missed potentially disease-associated CNVs. Future studies using short- or long-read genome sequence will provide a more comprehensive dataset that includes other variant types (*e.g.* repeat expansions, epigenetic changes) that also contribute. Many of the CNVs we identified – even those that are clear risk factors – do not fully segregate with disease. Comprehensive evaluation of exome sequence data in families with risk-conferring CNVs may reveal combinatorial genetic factors in some individuals, and it is likely that larger cohorts will be required to understand the complex genetic landscape of the common epilepsies.

## Supplementary Material

Supinfo2

Supinfo1

## Figures and Tables

**Table 1:** Summary of validated CNVs likely associated with disease risk

	Pairs (n = 859)	Multiplex Families (n = 267)	Total
Recurrent risk-associated CNVs	23	7	30
CNVs involving epilepsy genes	3	2	5
Large CNVs (>500 kb)	3	5	8
Total	29	14	43

**Table 2:** CNVs likely or potentially contributing to epilepsy risk

Likely Pathogenic CNVs: Pairs						
Family	Epilepsy type (proband)	Comorbidities (proband)	CNV	Size	CNV in family member? (epilepsy type & cormorbidities)	Genes
amq	GGE	None	22q12.2 del	~150 Kb	yes (NAFE)	*DEPDC5*
avu	NAFE	SD	22q12.2 dup	~ 30 Kb	yes (UNC)	*DEPDC5*
bax	NAFE	Cortical dysplasia in right frontal lobe, SD, ID	22q12.2 del	~ 10 Kb	yes (UNC)	*DEPDC5*
**Likely Pathogenic: Multiplex Families**						
cif	EOAE	ID,*	1q43 del	~320 kb	*de novo*	*COX20, HNRNPU, EFCAB2*
ckx	FLE	ID	22q12.2 del	~100 kb	father (unaffected); brother (FLE)	*DEPDC5, PRR14L*
**Recurrent CNVs associated with epilepsy risk: Pairs**						
ayq1	NAFE	ID	16p11.2 dup	~500 Kb	yes (NAFE, ADD)	
arz1	GGE	None	16p11.2 dup	~500 Kb	yes (UNC)	

azv1	NAFE	None	16p13.11 del	~1.5 Mb	yes (NAFE, **)	
aqz1	GGE	None	16p13.11 del	~1.5 Mb	yes (MIX)	
aes1	GGE	None	16p13.11 del	~1.5 Mb	yes (GGE)	
aec1	UNC	None	16p13.11 del	~1.5 Mb	yes (MIX, SD)	
bmv1	GGE	ID	16p13.11 del	~2.6 Mb	yes (GGE, ID)	
bae1	GGE	None	16p13.11 del	~1.5 Mb	no	
aru1	GGE	None	16p13.11 del	~1.5 Mb	no	
acu1	GGE	None	16p13.11 del	~ 3 Mb	no	

akj1	GGE	ID	15q11.2 del	~228 Kb	yes (GGE)	
ata1	GGE	None	15q11.2 del	~228 Kb	yes (UNC)	
adr1	GGE	None	15q11.2 del	~228 Kb	yes (GGE)	
adx1	GGE	None	15q11.2 del	~228 Kb	yes (GGE, ADHD)	
acl1	GGE	None	15q11.2 del	~228 Kb	no	
aym1	NAFE	None	15q11.2 del	~228 Kb	no	
aml2	Mixed	None	15q11.2 del	~228 Kb	no	

ami1	GGE	ID	15q13.3. del (BP4-BP5)	~4 Mb	yes (NAFE)	
vf1	GGE	**	15q13.3. del (BP4-BP5)	~1.5 Mb	yes (GGE)	
awm1	NAFE	Bilateral deep white matter microangiopathy lesions	15q13.3. del (BP4-BP5)	~1.5 Mb	no	

wj1	GGE	ID, SD	1q21.1 dup	~400 Kb	yes (MIX, SD, ID)	
aro1	GGE	ID	1q21.1 dup	~400 Kb	no	

bia	NAFE	ADHD	15q11 dup	~5 Mb	yes (NAFE, ID)	
Recurrent CNVs associated with epilepsy risk: Multiplex Families						
Family	Phenotype		CNV	Size	Segregation	
bit	NAFE	None	15q11.2 del	~500 Kb	father (affected)	
btk	MAE	ID	15q11.2 del	~500 Kb	*de novo*	
cie	EOAE	None	15q11.2 del	~500 Kb	father (unaff)	
cfu	GGE	None	15q11.2 del		not tested	

aap	UNC	None	15q13.3 del	~1.6 Mb	daughter (GGE, ID)	
ckl	GGE	None	15q13.3 del	~ 1.6 Mb	mother (GGE) mat aunt (unaff)	

chv	CAE/JAE	None	16p13.11 del	~1.2 Mb	daughter (EOAE, ID) sister (UNC) brother (GGE)	
**Variants >500kb of Unproven Pathogenicity: Multiplex Families**						
cio	GGE	None	18q del	~ 7.5 Mb	father (unaff)	*CDH7, CDH19, MR5011, DSEL, TMX3, DPC6, DOC6, TTN, SOCS6, CTSCR1, CBLN2, METO1*
cjd	NAFE	None	2q24.3 dup	~ 500 Kb	father (unaff)	*SCN1A, CALNT3, TT2C1B, SCN9A*
bec	unaffected father	None	15q13 dup (BP4–5)	~ 1.6 Mb	not found in affected child (GGE)	*APHGAP11B, HERC2P10, FAN1, MTMR10, TRPM1, KLF13, OTUD7A, CHRNA7*
cjw	NAFE	None	15q13 dup (BP4–5)	~ 1.6 Mb	brother (NAFE)	*APHGAP11B, HERC2P10, FAN1, MTMR10, TRPM1, KLF13, OTUD7A, CHRNA7*
ckx	FLE	ID	15q13.2 dup	~1 Mb	father (unaffected); brother (FLE)	*CHRNA7*
**Variants >500kb of Unproven Pathogenicity: Pairs Cohort**						
ve	UNCLASS.	None	9q34.3 del	~600 Kb	yes (mixed, ID)	*KCNT1*
bcm	NAFE	ADD	20p12.2 del	~ 1 Mb	no	*PLCB1, PLCB4*
uf	GGE	ID	18q21.2 dup	~20 Kb	yes (GGE, OCD)	*TCF4*

GGE, genetic generalized epilepsy; NAFE, non-acquired focal epilepsy; UNC, unclassified epilepsy; EOAE, early onset absence epilepsy; CAE, childhood absence epilepsy; JAE, juvenile absence epilepsy; FLE, frontal lobe epilepsy ID, intellectual disability; SD, speech delay; OCD, obsessive compulsive disorders: ADD, attention deficit disorder; ADHD, attention deficit hyperactivity disorder;

*, regressed in teens – severe ID as an adult;

**, head injury prior to onset of seizures

## Data Availability

Exome data is available in dbGaP for the research participants who consented to data sharing.

## Box

We confirm that we have read the Journal’s position on issues involved in ethical publication and affirm that this report is consistent with those guidelines.

- We investigate the role of rare copy number variants (CNVs) in common, familial epilepsies.
- Exome data can be used to predict both rare and common CNVs that may contribute to disease risk or etiology.
- Large, recurrent deletions contribute to epilepsy risk in 2.5–3% of families with generalized or focal epilepsy with incomplete penetrance.
- The overall CNV landscape in common familial epilepsies is similar to sporadic epilepsies.

## APPENDIX: Epi4K Consortium collaborators

**Table:**

Name	Affiliation(s)
***Core Analysis Group***	
Edith Almanza Fuerte	Center for Pediatric Neurological Disease Research, St. Jude Children’s Research Hospital, Memphis, TN 38105, USA
John Nguyen	Department of Pediatrics, Division of Genetic Medicine, University of Washington, Seattle, Washington 98195, USA.
Michelle Mehaffey	Department of Pediatrics, Division of Genetic Medicine, University of Washington, Seattle, Washington 98195, USA.
Arvis Sulovari	Department of Genome Sciences, University of Washington, Seattle, WA
Tianyun Wang	Department of Genome Sciences, University of Washington, Seattle, WA
Miranda Galey	Department of Pediatrics, Division of Genetic Medicine, University of Washington, Seattle, Washington 98195, USA.
Danny E. Miller	Department of Pediatrics, Division of Genetic Medicine, University of Washington, Seattle, Washington 98195, USA.
Evan E. Eichler	Department of Genome Sciences, University of Washington, Seattle, WA 98195, USA. Howard Hughes Medical Institute, University of Washington, Seattle, WA 98195, USA.
Heather C. Mefford	Center for Pediatric Neurological Disease Research, St. Jude Children’s Research Hospital, Memphis, TN 38105, USA
	
***Epi4K Consortium & EPGP Members***	
Bassel Abou-Khalil	Department of Neurology, Vanderbilt University Medical Center, Nashville, Tennessee 37232 USA.
Zaid Afawi Afawi	Ben-Gurion University of the Negev, Beer-Sheva, Israel and Erasmus MC, Rotterdam, the Netherlands.
Andrew S. Allen	Center for Statistical Genetics and Genomics, Department of Biostatistics and Bioinformatics, Duke University, Durham, North Carolina 27710, USA.
Dina Amrom	Departments of Neurology, Neurosurgery, McGill University and Neurogenetics Unity, Montreal Neurological Institute & Hospital, Montreal, Quebec H3A 2B4 Canada; Pediatric Neurology Unit, Centre Hospitalier de Luxembourg, Grand-Duchy of Luxembourg
Eva Andermann	Departments of Neurology, Neurosurgery and Human Genetics, McGill University, Montreal, Quebec H3A 2B4 Canada.
Jocelyn F. Bautista	Department of Neurology, Cleveland Clinic Lerner College of Medicine, Epilepsy Center of the Cleveland Clinic Neurological Institute, Cleveland, Ohio 44195 USA.
Susannah T. Bellows	Epilepsy Research Centre, Department of Medicine, University of Melbourne (Austin Health), Heidelberg, Victoria 3084, Australia.
Samuel F. Berkovic	Epilepsy Research Centre, Department of Medicine, University of Melbourne, Austin Health, Heidelberg, Victoria, Australia.
Judith Bluvstein	Department of Neurology, Zucker Hofstra School of Medicine, Lenox-Hill Hospital, New York, NY 10075.
Alexis Boro	Department of Neurology, Montefiore Medical Center, Albert Einstein College of Medicine, Bronx, New York, 10467 USA.
Rosemary Burgess	Epilepsy Research Centre, Department of Medicine, University of Melbourne (Austin Health), Heidelberg, Victoria 3084, Australia.
Gregory D. Cascino	Division of Epilepsy, Mayo Clinic, Rochester, Minnesota 55905 USA.
Seo-Kyung Chung	Neurology and Molecular Neuroscience Research, Institute of Life Science, Swansea University Medical School, Swansea University, Swansea SA2 8PP, UK; Kids Neuroscience Centre, Kids Research, Children’s Hospital at Westmead, Sydney, NSW 2145, Australia.
Damian Consalvo	Epilepsy Section, Neurology Division, Ramos Mejía Hospital, Buenos Aires, 1221, Argentina.
Patrick Cossette	Département de neurosciences, Université de Montréal, Centre Hospitalier de l’Université de Montréal, Quebec, Canada.
Douglas E. Crompton	Department of Neurology, Northern Health, Epping, Victoria 3076, Australia; Epilepsy Research Centre, Department of Medicine, University of Melbourne (Austin Health), Heidelberg, Victoria 3084, Australia.
Patricia Crumrine	Medical Epilepsy Program & EEG & Child Neurology, Children’s Hospital of Pittsburgh of UPMC, Pediatrics, University of Pittsburgh School of Medicine, Pittsburgh, Pennsylvania 15224 USA.
Sarah W. Curtis	Emory
Norman Delanty	Department of Neurology, Beaumont Hospital, and FutureNeuro Research Centre, Royal College of Surgeons in Ireland, Dublin 9, Ireland.
Orrin Devinsky	NYU Comprehensive Epilepsy Center, New York University, Department of Neurology, NYU School of Medicine, New York, New York 10016, USA.
Dennis Dlugos	Department of Neurology and Pediatrics, The Children’s Hospital of Philadelphia, Perelman School of Medicine at the University of Pennsylvania, Philadelphia, Pennsylvania 19104, USA.
Colin A. Ellis	Department of Neurology, Perelman School of Medicine at the University of Pennsylvania, Philadelphia, Pennsylvania 19104, USA.
Michael P. Epstein	Department of Human Genetics, Emory University School of Medicine, Atlanta, Georgia, 30322, USA.
Miguel Fiol	Department of Neurology, Epilepsy Care Center, University of Minnesota Medical School, Minneapolis 55414 USA.
Nathan B. Fountain	FE Dreifuss Comprehensive Epilepsy Program, University of Virginia, Charlottesville, Virginia 22908 USA.
Catharine Freyer	Department of Neurology, University of California, San Francisco, San Francisco, California 94143 USA.
Dan Friedman	Department of Neurology, New York University, Grossman School of Medicine, New York, NY, USA
Eric B. Geller	Institute of Neurology and Neurosurgery at Cooperman Barnabas Medical Center, Saint Barnabas Medical Center, Livingston NJ, USA.
Tracy Glauser	Division of Neurology, Cincinnati Children’s Hospital Medical Center, Cincinnati, Ohio 45229 USA.
Simon Glynn	Department of Neurology and Neuroscience Graduate Program, University of Michigan Medical Center, Ann Arbor, and Ann Arbor Veterans Administration Healthcare System, Ann Arbor, Michigan, USA.
David B. Goldstein	Institute for Genomic Medicine, Columbia University Medical Center, New York, New York 10032 USA.
Micheline Gravel	Centre of Excellence in Neuromics and CHUM Research Center, Université de Montréal, Quebec, H2X 0A9, Canada
Kevin Haas	Department of Neurology, Vanderbilt University Medical Center, Nashville, Tennessee 37232 USA.
Rebekah V. Harris	Epilepsy Research Centre, Department of Medicine, University of Melbourne (Austin Health), Heidelberg, Victoria 3084, Australia.
Sheryl Haut	Department of Neurology, Montefiore Einstein, 111 East 210th St, Bronx, NY 1046.
Erin L. Heinzen	Division of Pharmacotherapy and Experimental Therapeutics, Eshelman School of Pharmacy, and Department of Genetics, School of Medicine, University of North Carolina, Chapel Hill, North Carolina, USA.
Sandra Helmers	Department of Neurology, Emory University Hospital, Atlanta, GA, USA.
Olivia J. Henry	Centre for Inherited Metabolic Diseases, Karolinska University Hospital, Stockholm, Sweden. Department of Molecular Medicine and Surgery, Karolinksa Institutet, Stockholm, Sweden
Sucheta Joshi	Department of Pediatrics, University of Michigan, Ann Arbor, Michigan 48109 USA.
Heidi E. Kirsch	Department of Neurology, University of California, San Francisco, San Francisco, California 94143 USA.
Sara Kivity	Epilepsy Unit, Schneider Children’s Medical Center of Israel, Petach Tikvah, Israel.
Robert C. Knowlton	Department of Neurology, University of California, San Francisco, San Francisco, California 94143 USA.
Eric Kossoff	Department of Neurology, Johns Hopkins Hospital, Baltimore, Maryland 21287 USA.
Ruben Kuzniecky	Department of Neurology, Zucker Hofstra School of Medicine, Lenox-Hill Hospital, New York, NY 10075.
Rebecca Loeb	Division of Gastroenterology and Hepatology, University of California, San Francisco, San Francisco, California 94143 USA.
Daniel H. Lowenstein	Department of Neurology, University of California, San Francisco, San Francisco, California 94143 USA.
Anthony G. Marson	Department of Pharmacology and Therapeutics, University of Liverpool, Clinical Sciences Centre, Lower Lane, Liverpool L9 7LJ, UK
Mark McCormack	Molecular & Cellular Therapeutics, Royal College of Surgeons in Ireland, Dublin 2, Ireland; and Department of Genetics, Universitair Medisch Centrum Utrecht, Utrecht, the Netherlands.
Shannon M. McGuire	Clinical Neurology, Children’s Hospital Epilepsy Center of New Orleans, New Orleans, Louisiana 70118 USA.
Kevin McKenna	Department of Neurology, University of California, San Francisco, San Francisco, California 94143 USA.
Heather C. Mefford	Center for Pediatric Neurological Disease Research, St. Jude Children’s Research Hospital, Memphis, TN, 38105, USA.
Paul V. Motika	Comprehensive Epilepsy Center, Oregon Health and Science University, Portland, OR 97239 USA.
Saul A. Mullen	Epilepsy Research Centre, Department of Medicine, University of Melbourne (Austin Health), Heidelberg, Victoria 3084, Australia
Edward J. Novotny	Departments of Neurology and Pediatrics, University of Washington School of Medicine, Seattle Children’s Hospital, Seattle, Washington 98105 USA.
Terence J. O’Brien	Department of Neuroscience, Central Clinical School, Alfred Health, Monash University, Melbourne, Victoria, AUSTRALIA.
Karen L. Oliver	Epilepsy Research Centre, Department of Medicine (Austin Health), The University of Melbourne, Heidelberg, VIC 3031, Australia
Ruth Ottman	G. H. Sergievsky Center, Columbia University, New York, NY 10032, USA; and Department of Neurology, Columbia University, New York, NY 10032, USA; and Department of Epidemiology, Columbia University; and Division of Translational Epidemiology and Mental Health Equity, New York State Psychiatric Institute, New York, NY 10032
Juliann M. Paolicchi	Department of Neurology and Pediatrics, Zucker Hofstra School of Medicine, SIUH and Lenox-Hill Hospital, New York, NY 10075
Jack M. Parent	Department of Neurology and Michigan Neuroscience Institute, Michigan Medicine, Ann Arbor, and Ann Arbor Veterans Administration Healthcare System, Ann Arbor, Michigan, USA.
Kristen L. Park	University of Colorado School of Medicine, Aurora CO 80045 USA
Sarah J. Paterson	Department of Paediatrics and Child Health, University of Otago, Wellington, New Zealand.
Slave Petrovski	Epilepsy Research Centre, Department of Medicine, University of Melbourne (Austin Health), Heidelberg, Victoria 3084, Australia.
William O. Pickrell	Wales Epilepsy Research Network, Swansea University Medical School, Swansea University, Wales, UK; and Neurology Department, Morriston Hospital, Swansea Bay University Health Board, Wales, UK.
Annapurna Poduri	Division of Epilepsy and Clinical Neurophysiology, Department of Neurology, Boston Children’s Hospital, Boston, Massachusetts 02115, USA.
Mark I. Rees	Faculty of Medicine & Health, University of Sydney, Sydney, Australia; Faculty of Medicine and Life Science, Swansea University, Swansea, Wales, UK
Lynette G. Sadleir	Department of Paediatrics and Child Health, University of Otago, Wellington, New Zealand.
Ingrid E. Scheffer	Epilepsy Research Centre, Department of Medicine, University of Melbourne (Austin Health), Heidelberg, Victoria 3084, Australia; and Florey Institute and Department of Paediatrics, Royal Children’s Hospital, Melbourne, Victoria, Australia.
Renee A. Shellhaas	Department of Neurology, Washington University School of Medicine, St. Louis, Missouri 63110 USA.
Elliott H. Sherr	Weill Institute of Neuroscience, Departments of Neurology, Pediatrics and Institute of Human Genetics, University of California, San Francisco, San Francisco, California 94158 USA.
Jerry J. Shih	Comprehensive Epilepsy Center, University of California San Diego, School of Medicine, La Jolla, CA, USA
Shlomo Shinnar	Albert Einstein College of Medicine, Bronx, NY.
Rani K Singh	Atrium health-Levine Children’s Hospital, USA.
Joseph Sirven	Department of Neurology, Mayo Clinic, Scottsdale, Arizona 85259 USA.
Michael C. Smith	Rush University Medical Center, Rush Epilepsy Center, Chicago, IL.
Philip E.M. Smith	Department of Neurology, University Hospital of Wales, Heath Park, Cardiff, Wales, CF14 4XW, UK.
Michael R. Sperling	Jefferson Comprehensive Epilepsy Center, Department of Neurology, Sidney Kimmel Medical College at Thomas Jefferson University
Joseph Sullivan	Department of Neurology, University of California, San Francisco, San Francisco, California 94143 USA
Liu Lin Thio	Department of Neurology, Washington University School of Medicine, St. Louis, Missouri 63110 USA.
Rhys H. Thomas	Translational and Clinical Research Institute, Faculty of Medical Sciences, Newcastle University, UK.
Anu Venkat	Department of Pediatrics, Children’s Hospital at Saint Peter’s University Hospital, Rutgers Robert Wood Johnson Medical School, New Brunswick, NJ, USA.
Eileen P. G. Vining	Department of Neurology, Johns Hopkins Hospital, Baltimore, Maryland 21287 USA.
Gretchen K. Von Allmen	Division of Child & Adolescent Neurology, Department of Pediatrics, McGovern Medical School and UTHealth, University of Texas at Houston, Houston, Texas 77030 USA.
Judith Weisenberg	Department of Neurology, Washington University School of Medicine, St. Louis, Missouri 63110 USA.
Peter Widdess-Walsh	Department of Neurology, Beaumont Hospital, Dublin, Ireland.
Melodie R. Winawer	G. H. Sergievsky Center, Columbia University, New York, NY 10032, USA; and Department of Neurology, Columbia University, New York, NY 10032, USA.
