# Supplementary material for: The role of copy number variants in the genetic architecture of common familial epilepsies
Source: Epilepsia. Author manuscript; Available in PMC 2025 Mar 1. (PMC10948303; doi:10.1111/epi.17860)

The role of copy number variants in the genetic architecture of common familial epilepsies

Epi4K Consortium

Supplemental Material – Pedigrees for Families with epilepsy risk-associated CNV.

SUPPLEMENTAL FIGURES:
Family pedigrees

Key

- Generalised epilepsy of unknown cause
- Photosensitivity
- Focal epilepsy of unknown cause
- Mixed generalised and focal epilepsy of unknown cause
- Unclassified epilepsy
- Isolated unprovoked seizure of unknown cause
- Symptomatic unprovoked seizure(s)
- Febrile seizure(s)
- Febrile seizures plus
- Acute seizure
- Unclassified event; unknown if provoked or unprovoked seizure, or unconfirmed seizure
- **B** Blood sample available

Family cif

De novo 1q44 deletion encompassing *HNRNPU* in proband (arrow). Upon review her phenotype is more severe, consistent with *HNRNPU*-disorder

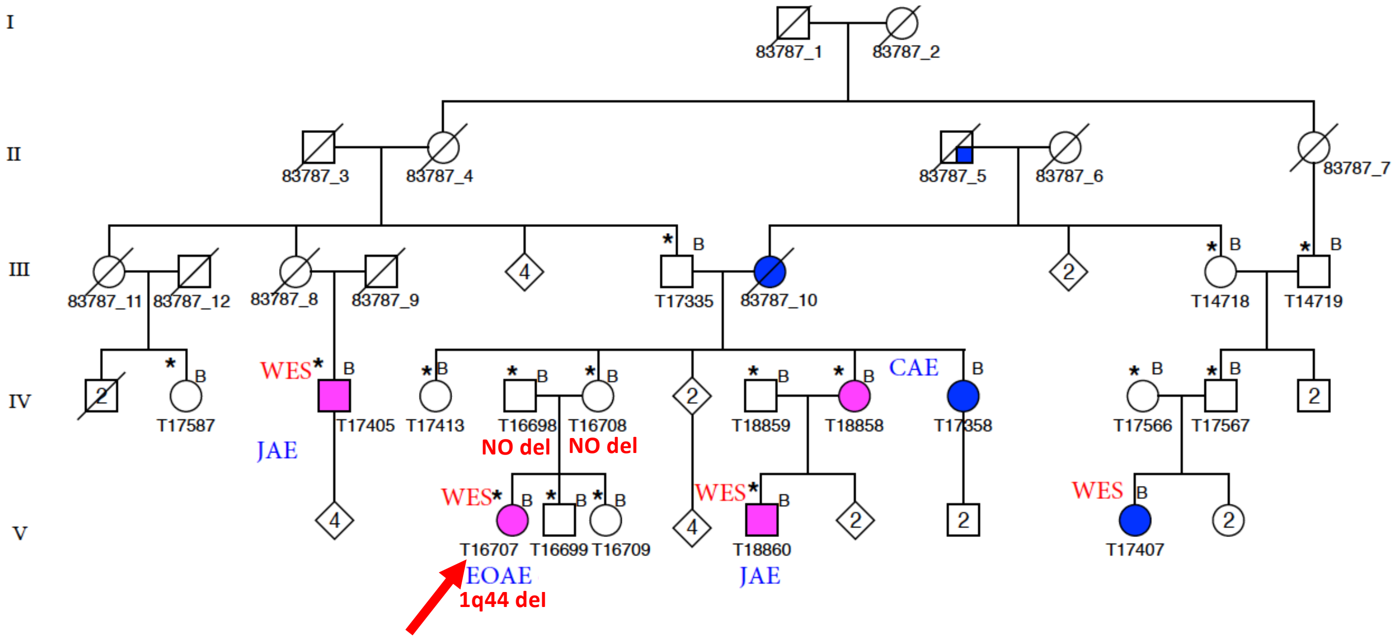

Family cio

7.5 Mb deletion of 18q22 (VUS) validated in proband (arrow) and his unaffected father who is mosaic. 18q syndrome usually has deleted terminal portion of 18q, not seen here.

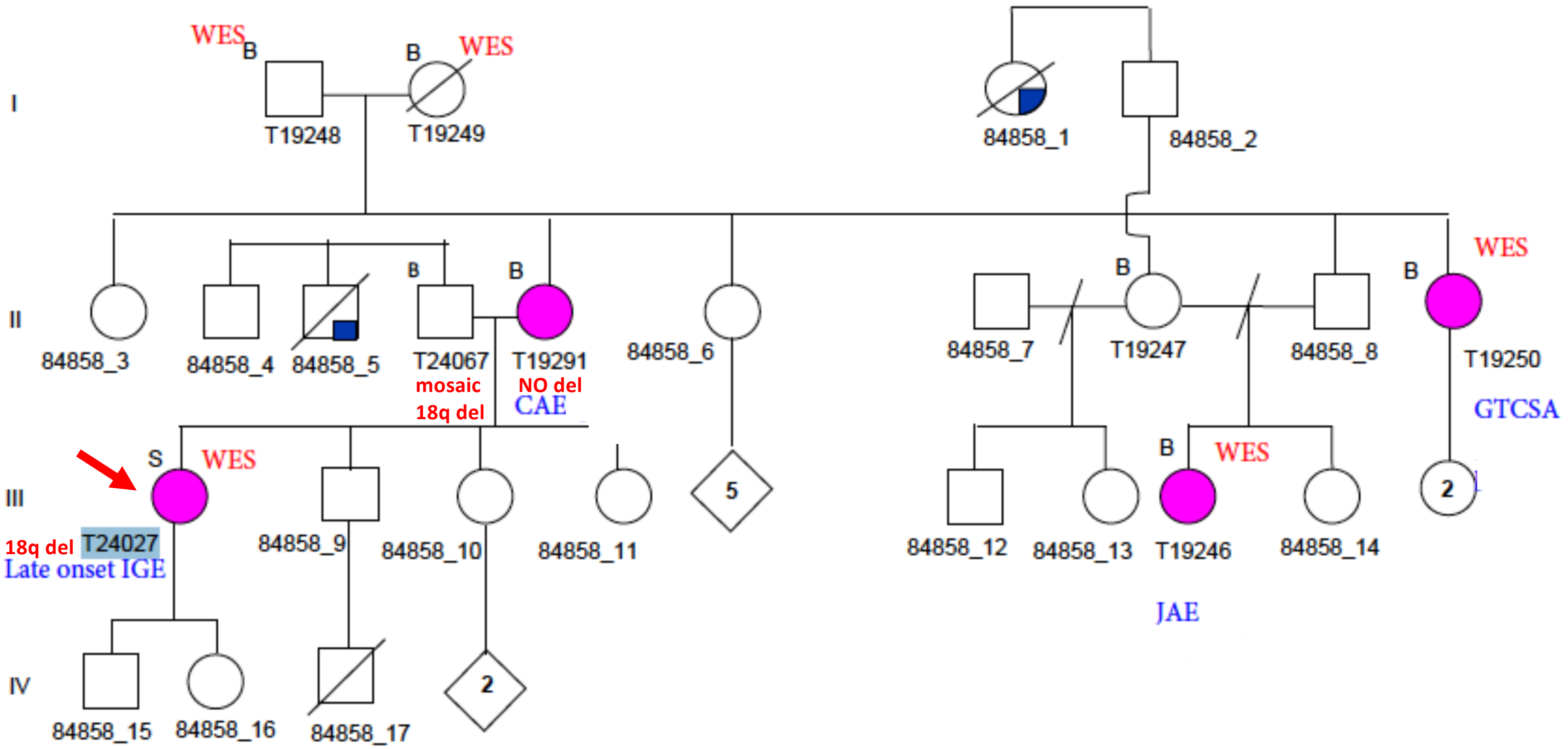

Family cjd

2q24 duplication (likely benign) encompassing *SCN1A* validated in proband, unaffected father. Duplication does not encompass *SCN3A* and/or *SCN2A.*

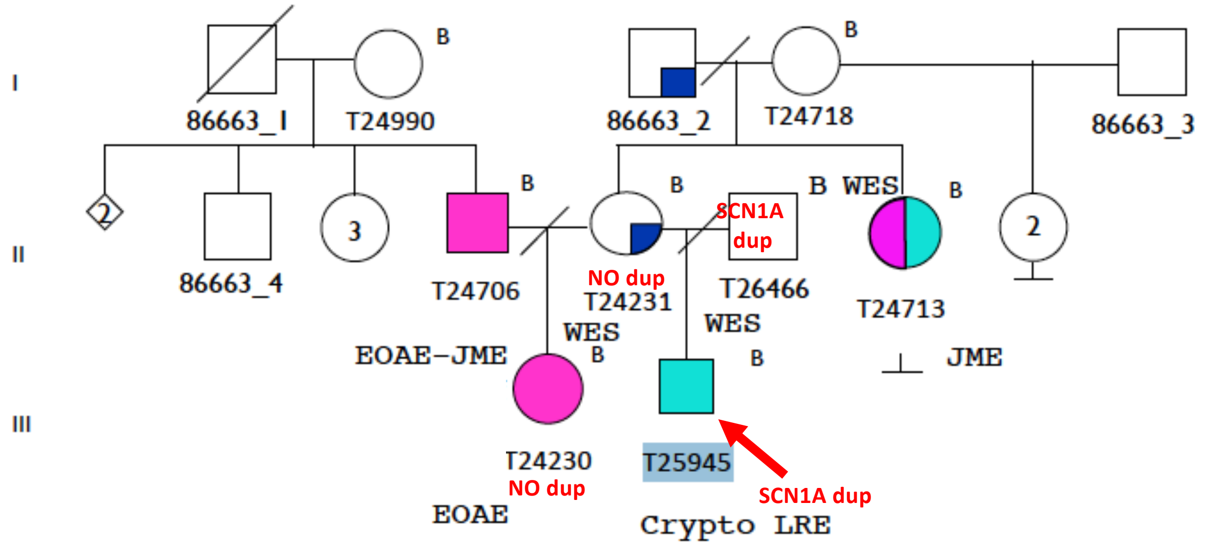

Family ckx
Intragenic *DEPDC5* deletion & *CHRNA7* duplication validated in proband (arrow), affected brother and unaffected father. Loss of function *DEPDC5* variants are known to cause focal epilepsies with incomplete penetrance.

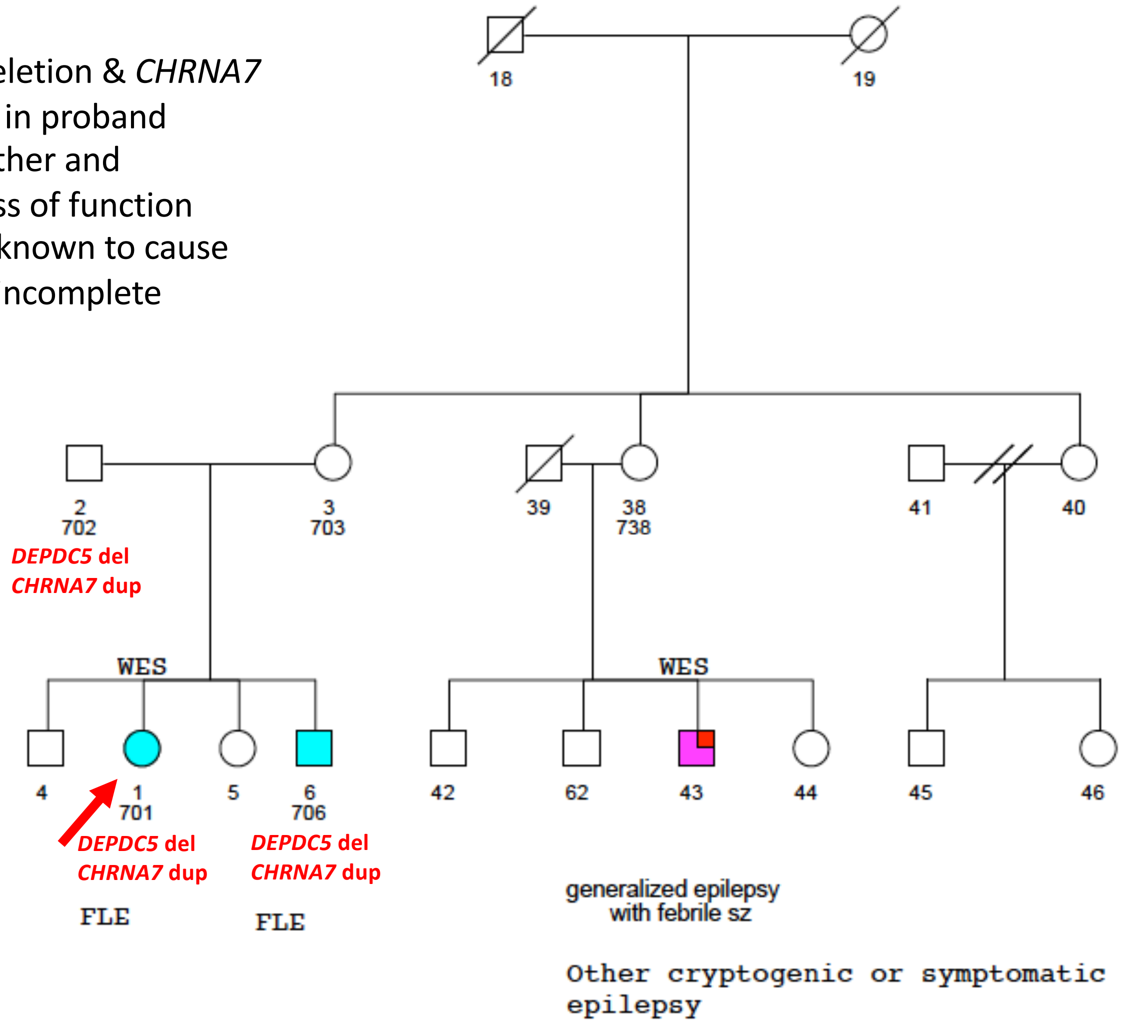

Family ckl
15q13 deletion (known recurrent CNV associated with risk of neurodevelopmental disorders) was validated in proband (arrow), mom (GGE), and maternal aunt (unaffected). Deletion was also called in cousin, but unable to validate as DNA was unavailable.

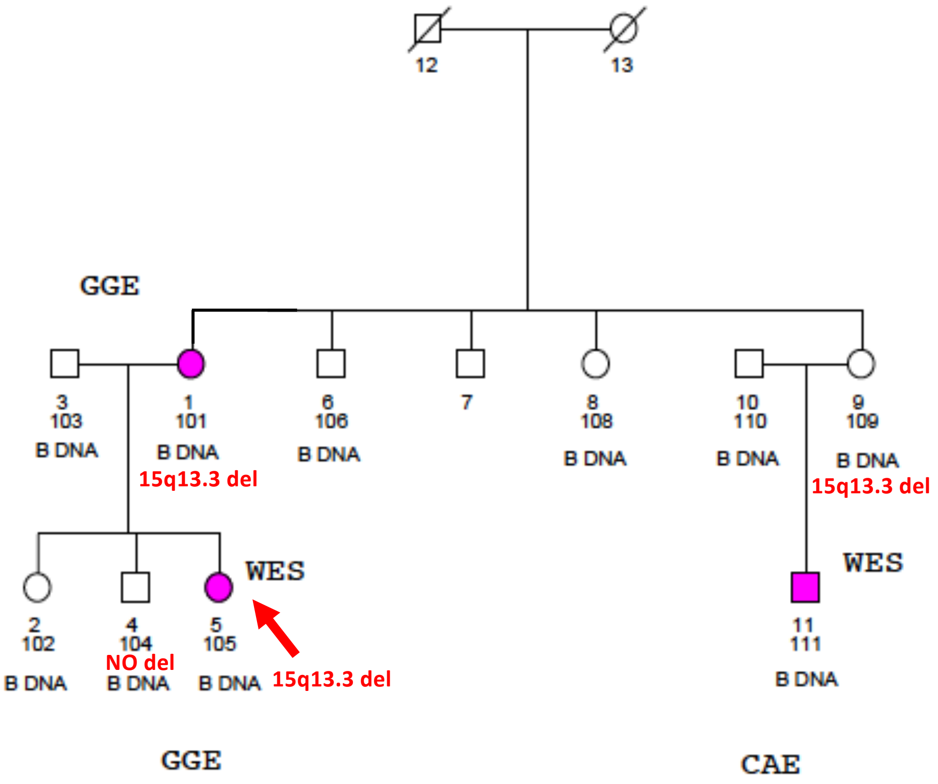

Family bec
15q13 BP4-BP5 dup (VUS). CNV validated in unaffected father (arrow), but not in either of his affected children.

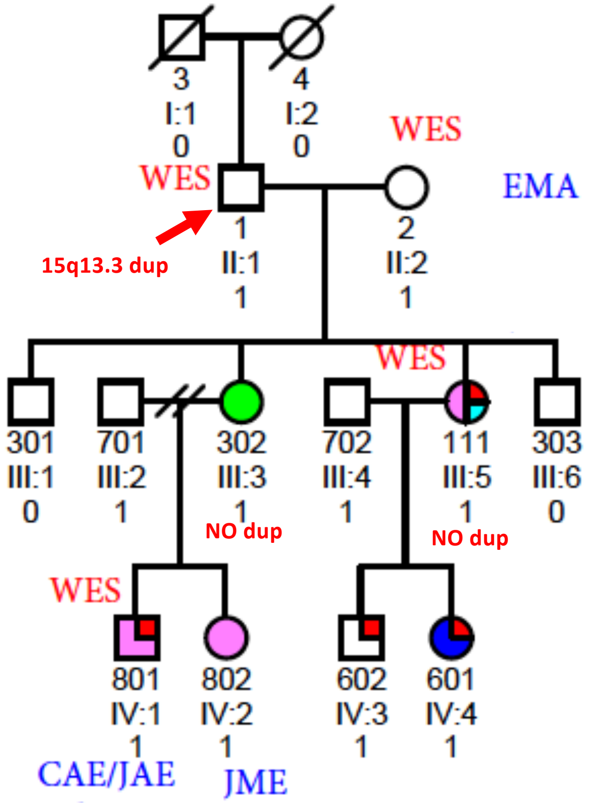

Family cjw
15q13 BP4-BP5 dup (VUS). CNV validated in proband (arrow) as well as brother (single unprovoked seizure) and not seen in unaffected mother.

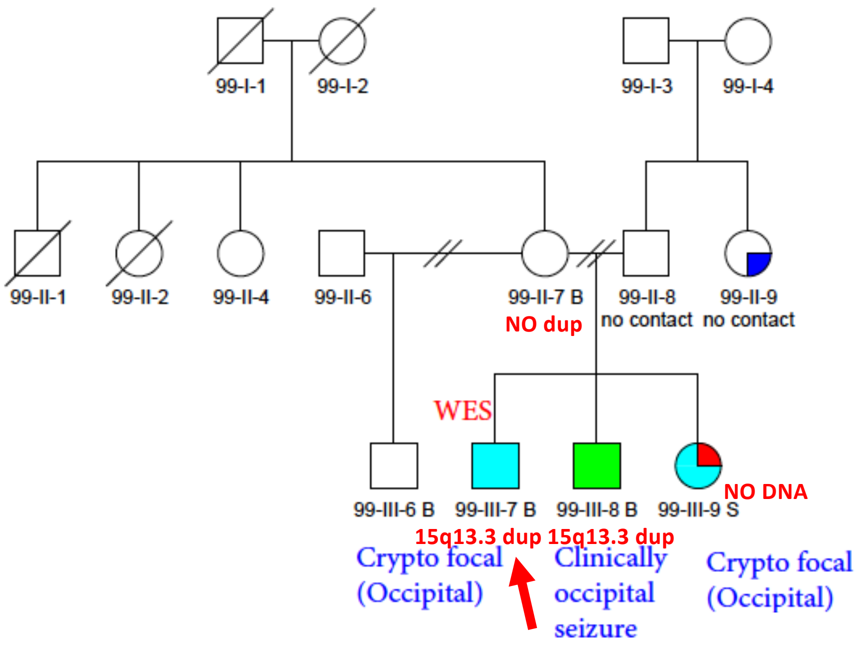

Family chv
16p13.11 del (known recurrent CNV associated with risk of neurodevelopmental disorders) segregated with seizure disorders in this family.

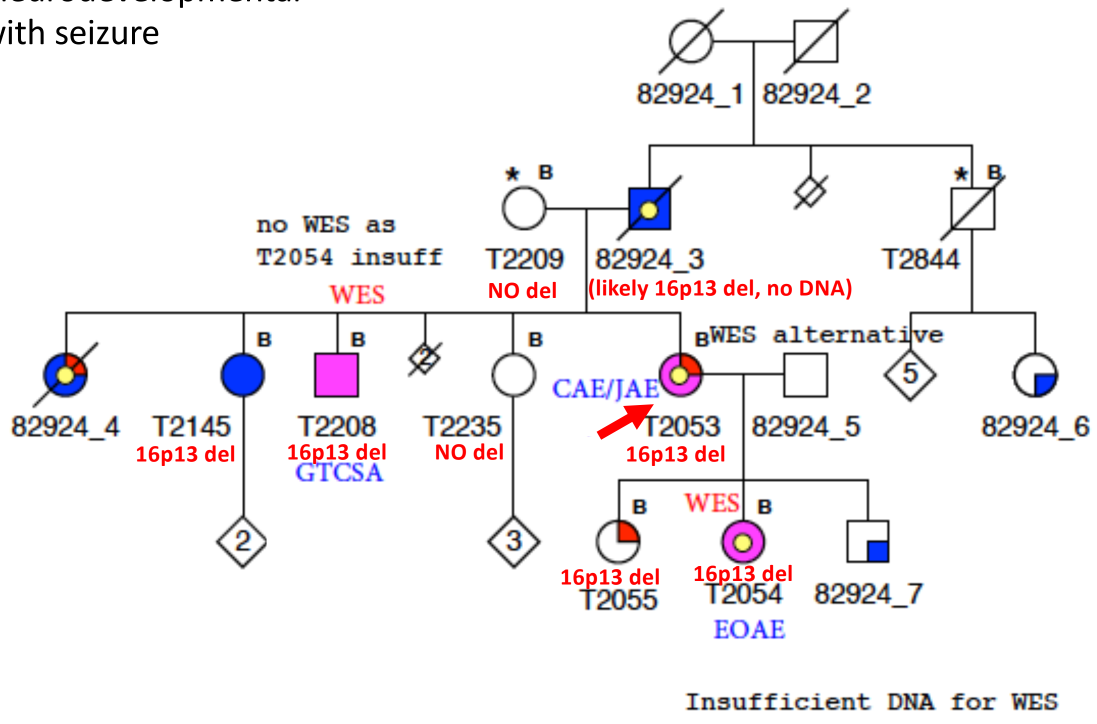

Family bit
15q11 del (known recurrent CNV associated with risk of neurodevelopmental disorders) validated in proband (arrow) as well as mother (UNC), but not daughter (GGE).

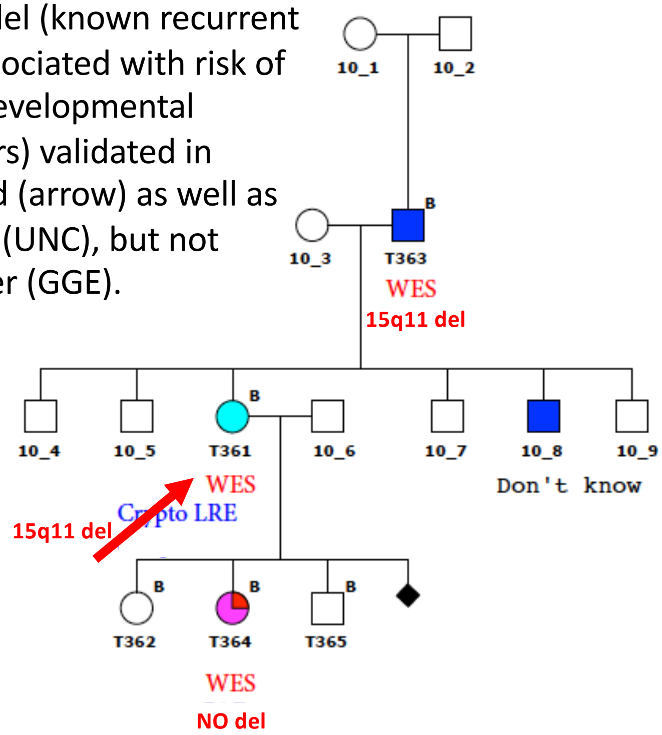

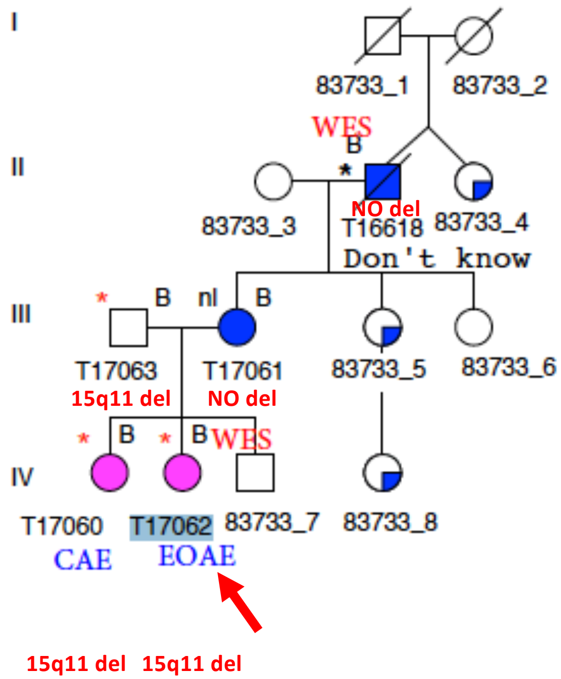

Family cie
15q11 del (known recurrent CNV associated with risk of neurodevelopmental disorders) validated in proband (arrow), sister (CAE), and unaffected father.

Family btk
15q11 del (known recurrent CNV associated with risk of neurodevelopmental disorders) *de novo* in proband (arrow).

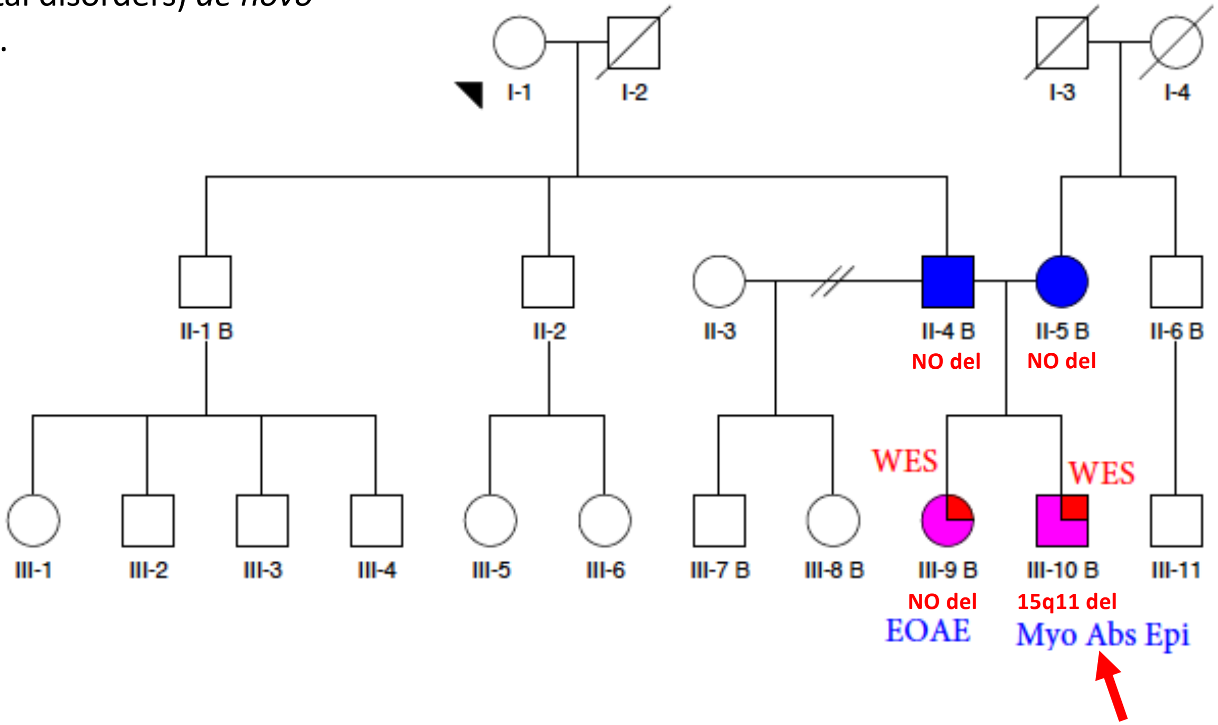

Family cfu
15q11 del (known recurrent CNV associated with risk of neurodevelopmental disorders) validated in proband (arrow). Family members not tested (DNA unavailable)

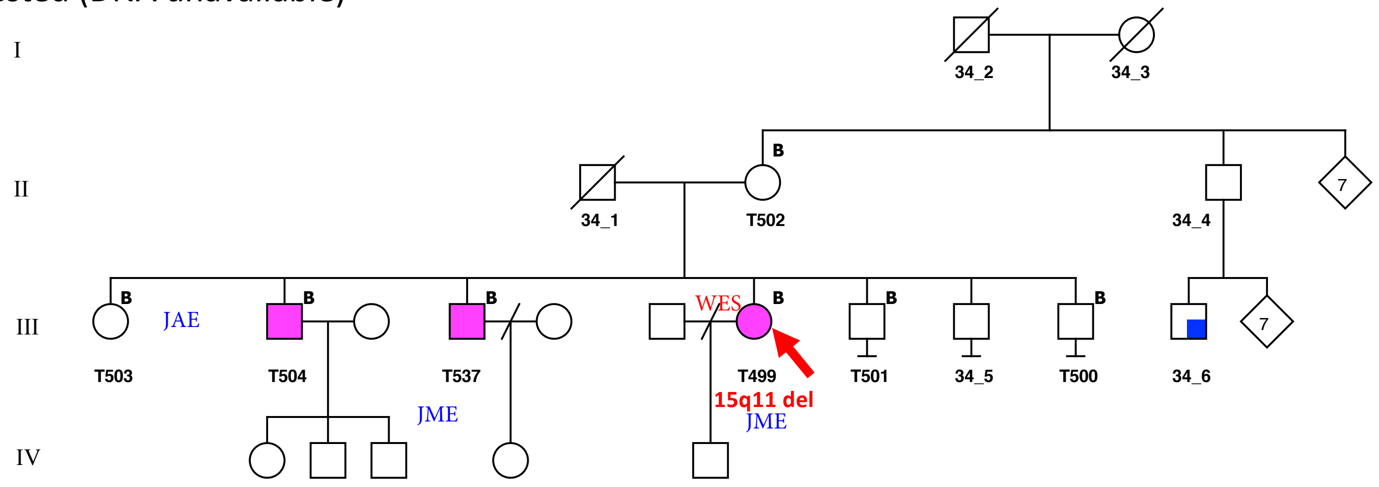

Individual	Onset Age	GTCS	MJ	Abs	AtypAbs	otherGen
T499	13y	Yes	Yes	No	No	No
T504	16y	No	No	Yes	No	No
T537	12y	Yes	Yes	No	No	No

Family cjw
15q13 dup (VUS) validated in proband and affected brother with occipital seizures.

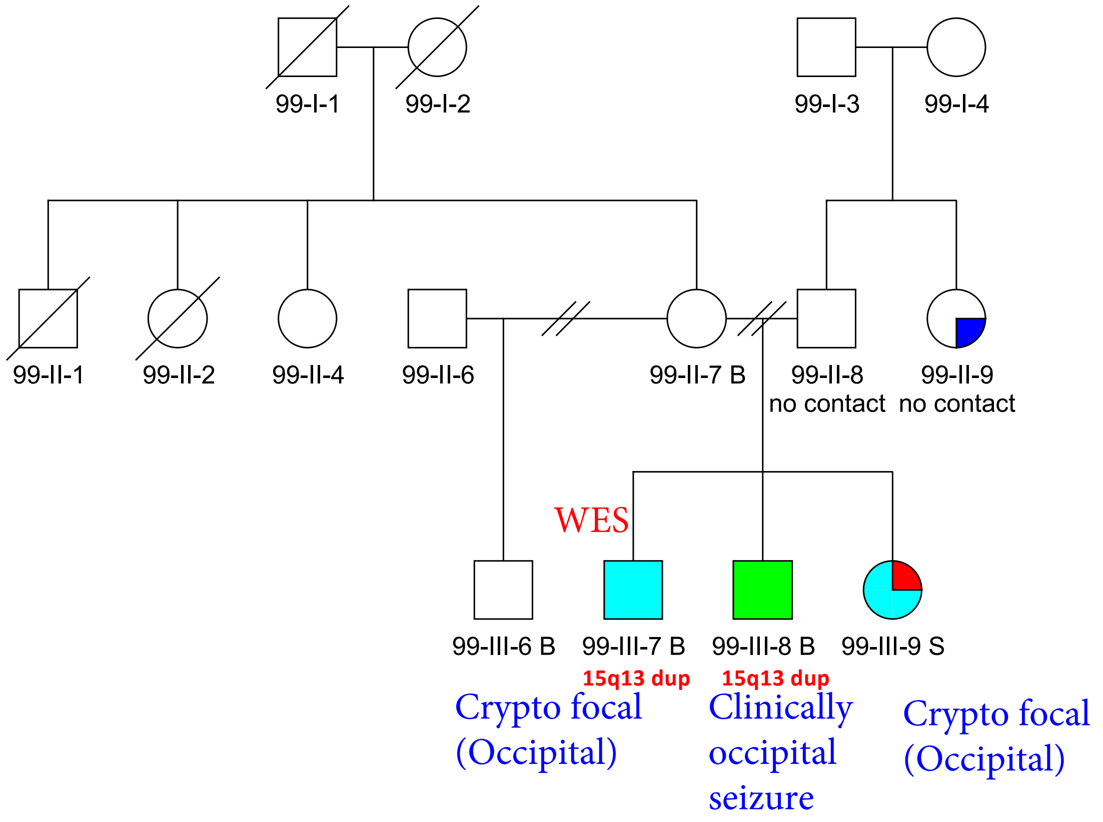

Family aap
15q11 del (known recurrent CNV associated with risk of neurodevelopmental disorders) validated in proband (arrow) and daughter (GGE).

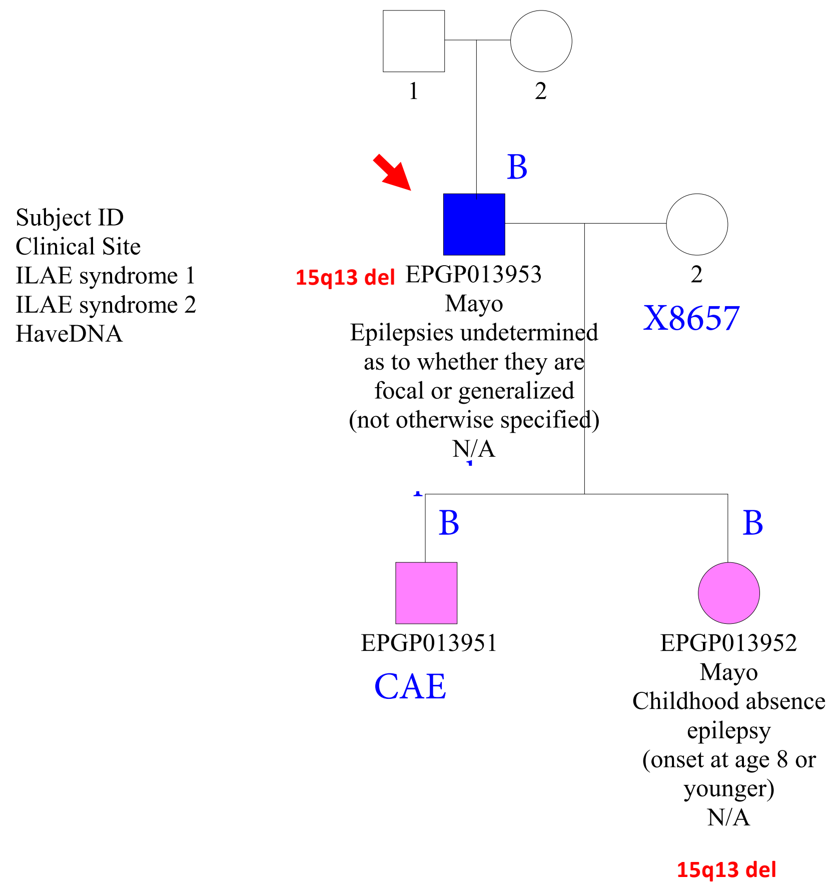

Supplement: Supinfo1 [file NIHMS1951868-supplement-Supinfo1.pdf]
